# Evaluation of plasma C-reactive protein levels in pregnant women with and without periodontal disease: A comparative study

**DOI:** 10.4103/0972-124X.60227

**Published:** 2009

**Authors:** Anupriya Sharma, Amitha Ramesh, Biju Thomas

**Affiliations:** *Department of Periodontics, Postgraduate Student, A.B. Shetty Memorial Institute of Dental Sciences, Mangalore, India*; 1 *A.B. Shetty Memorial Institute of Dental Sciences, Mangalore, India*; 2 *A.B. Shetty Memorial Institute of Dental Sciences, Mangalore, India*

**Keywords:** C-reactive protein, periodontal disease, preterm

## Abstract

**Background and Objectives:**

Circulating C-reactive protein (CRP) levels are a marker of systemic inflammation and are associated with periodontal disease, a chronic bacterial infection associated with elevation of proinflammatory cytokines and prostaglandins. CRP has been associated with adverse pregnancy outcomes, including preterm delivery, preeclampsia, and intrauterine growth restriction. Furthermore, periodontal disease has been associated with increased risk of preterm low birth weight, low birth weight, and preterm birth. The present study was conducted to assess plasma CRP levels in pregnant women with and without periodontal disease; to evaluate the effect of periodontal therapy on the incidence of preterm delivery; and to compare the incidence of preterm delivery in pregnant women with and without periodontal disease.

**Materials and Methods:**

A total of 90 pregnant women aged between 18-35 years with gestational age between 12-28 weeks were recruited and divided into three equal groups (control group, study group, treatment group) of 30 each. Blood samples were taken for estimation of C-reactive protein levels from all groups at 12-20 weeks of gestation, determined using ultrasensitive turbidimetric immunoassay (QUANTIA-CRP US). The treatment group comprised plaque control, scaling, and root planning and daily rinsing with 0.2% chlorhexidine mouth before 28 weeks of gestation.

**Results:**

The mean value of C-reactive protein levels in subjects with periodontal disease was higher compared to control group i.e., 1.20 ± 0.247 mg/dl and 1.22 ± 0.250 mg/dl, respectively, compared to 0.713 ± 0.139 mg/ dl (*P* = 0.001). The mean value of CRP levels before treatment was greater than the mean value after treatment i.e., 1.22 ± 0.25 compared to 0.84 ± 0.189 (*P* < 0.001). The incidence of preterm delivery (< 37 weeks) was 31.7% in the periodontal disease group (study group) compared to 8.3% in the control group (*P* = 0.001). The incidence of preterm delivery in the treatment group was 15.0% compared to 31.7% in the nontreatment group (study group).

**Conclusion:**

The findings from the study suggest that periodontal disease in pregnant women is associated with increased C- reactive protein levels in early pregnancy, incidence of preterm delivery is higher in pregnant women with periodontal disease compared to healthy controls, periodontal therapy during pregnancy reduces plasma CRP levels and there is decrease in incidence of preterm delivery after periodontal therapy.

## INTRODUCTION

C-reactive protein (CRP) is an acute phase reactant produced by liver in response to diverse inflammatory stimuli, including heat, trauma, infection, and hypoxia.[1] Circulating CRP levels are a marker of systemic inflammation and are associated with periodontal disease, a chronic bacterial infection associated with elevation of proinflammatory cytokines and prostaglandins. CRP has been associated with adverse pregnancy outcomes, including preterm delivery,[2] preeclampsia,[3] and intrauterine growth restriction.[4] Furthermore, periodontal disease has been associated with increased risk of preterm low birth weight,[5] low birth weight,[6] and preterm birth.[7] Preterm birth is a major medical, social, and economic problem accounting for large proportion of maternal and especially, neonatal mortality and morbidity. Preterm infants are at elevated risk for death, neurodevelopment disabilities, cognitive impairment, and behavioral disorders. Therefore, CRP might be a plausible mediator of the association between periodontitis and adverse pregnancy outcomes. Elevated CRP could amplify the inflammatory response through complement activation, tissue damage, and induction of inflammatory cytokines in the monocytes and therefore may mediate the relationship between periodontitis and adverse pregnancy outcomes.[8] Alternatively, periodontal disease and CRP may share a common risk factor predisposing certain individuals to a hyper inflammatory response.

### Objectives

This study was conducted with the following aims:

1. To assess plasma CRP levels in pregnant women with and without periodontal disease.
2. To evaluate the effect of periodontal therapy on the incidence of preterm delivery.
3. To compare the incidence of preterm delivery in pregnant women with and without periodontal disease.

## MATERIALS AND METHODS

### Source of data

The study was designed as a prospective cohort study involving a sample size of 90 pregnant women, reporting to the Department of Periodontics, A. B. Shetty Memorial Institute of Dental Sciences and Department of Obstetric and Gynaecology, K. S. Hedge Medical Academy, Deralakatte, Mangalore, divided into three equal groups of 30 each: Group I: Control group, periodontally healthy pregnant women; group II: Study group, pregnant women diagnosed clinically as suffering from periodontal disease; group III: Treatment group, pregnant women diagnosed clinically as suffering from periodontal disease and periodontal therapy was given in second trimester.

Screening examination included: 1) medical history; 2) obstetric history; 3) dental history; 4) Russell's periodontal index.

### Criteria for selection

#### Inclusion criteria

1. Subjects with age group between 18-35 years.
2. Subjects in good state of health without systemic disease.
3. Subjects with gestational age between 12-28 weeks.
4. Subjects were classified according to following criteria:
  - Simple gingivitis (Russell's Periodontal Index Score: 0.3-0.9).
  - Beginning destructive periodontal disease. (Russell's Periodontal Index Score: 0.7-1.9).
  - Established destructive periodontal disease (Russell's Periodontal Index Score: 1.7-5.0).
5. Subjects with minimum of 20 sound permanent teeth.

#### Exclusion criteria

1. Subjects with systemic disease.
2. Subjects with history of antibiotic intake during pregnancy.
3. Subjects with multiple pregnancies.
4. Subjects with diabetes prior to pregnancy.
5. Subjects with intention to deliver at hospital other than that of the study.
6. Other obstetric risk factors like smoking, alcohol consumption, drugs use, etc.

Periodontal disease activity was recorded at baseline for all groups using mouth mirror and UNC-15 probe. Blood samples were taken for estimation of C-reactive protein levels from all groups at 12-20 weeks of gestation. C-reactive protein levels were determined using ultrasensitive turbidimetric immunoassay (QUANTIA-CRP US), with a detection limit of 0.015 mg/dl. Periodontal therapy was done in the treatment group (Group III) consisting of plaque control, scaling and root planning, and daily rinsing with 0.2% chlorhexidine mouth before 28 weeks of gestation. Maintenance therapy was done in the control group (Group I) consisting of oral hygiene instructions and in the treatment group (Group III) consisting of oral hygiene instructions and supragingival plaque removal by instrumentation, as needed given every 2-3 weeks until delivery. Patients not maintaining oral hygiene in group I and group III were excluded from study. Incidence of preterm was compared in all groups.

### Data analysis

The data obtained was subjected to statistical analysis using two sample *t*-test, paired *t*-test to compare C-reactive protein values in group III before and after treatment, unpaired *t*-test to compare C-reactive protein values for term and preterm cases in each group, chi-square test to compare the incidence of preterm in different groups, analysis of variance (ANOVA) to determine difference in age and C-reactive protein values in different groups.

## RESULTS

In this study, an attempt was made to evaluate the CRP levels in pregnant women with and without periodontal disease and healthy control using ultrasensitive measurement of CRP, with a detection limit of 0.015 mg/dl. There was a statistically significant difference in mean CRP levels in different groups (*P* = 0.001) [Table 1a]. To further compare which particular pairs of groups were statistically significant, a post-hoc comparison test was carried out. There was a significant difference in group 1 and group 2, group 1 and group 3 (*P* <. 001) but there was no significant difference between group 2 and group 3 (*P* > 0.05) [Table 1b]. Post-hoc pairwise comparison of adjusted mean showed a significantly higher mean CRP values (1.204 and 1.22) in periodontal disease groups compared to control group (0.713) [Table 1c]. A statistically significant difference was observed in mean value of CRP plasma levels (*P* <. 001) in group III before and after treatment [Table 2]. The mean values of CRP levels in group I subjects with preterm delivery was 0.912 ± 0.697 (mean ± S.D) and term delivery 0.674 ± 0.113 (mean ± S.D). A significant difference was found in mean values of CRP levels (*P* <.001) in preterm and term delivery in group I subjects.

**Table 1a:** Comparison of mean C-reactive protein levels in different groups (descriptives)

C-RP	N	Mean	Std. deviation	Std. error
Group				
1	30	0.7137	0.13935	0.02544
2	30	1.2040	0.24784	0.04525
3	30	1.2257	0.25007	0.04566
Total	90	1.0478	0.32131	0.03387

F = 52.67 (One Way ANOVA); *P* = .001 HS

**Table 1b:** Post hoc tests (multiple comparisons)

Dependent variable C-RP Tukey HSD			Mean difference		95% confidence interval	
		
(I) Group	(J) Group	(I-J)	Std. error	*P* value	Lower bound	Upper bound
1	2	−0.49033*	0.05645	0.000	−0.6249	−0.3557
	3	−0.51200*	0.05645	0.000	−0.6466	−0.3774
2	1	0.49033*	0.05645	0.000	0.3557	0.6249
	3	−0.02167	0.05645	0.922	−0.1 563	0.1129
3	1	0.51200*	0.05645	0.000	0.3774	0.6466
	2	0.02167	0.05645	0.922	−0.1129	0.1563

* The mean difference is significant at the. 05 level.

**Table 1c:** Homogeneous subsets

C- RP			

Group	N	Subset for alpha = 0.05	
		
		1	2
1	30	0.71 37	
2	30		1.2 040
3	30		1.2257

Means for groups in homogeneous subsets are displayed.

**Table 2:** CRP levels in group III before and after periodontal therapy (paired *t*-test)

C-RP	Mean ± S.D	Std. error mean	Mean difference	95% CI of the difference	*t*	*P* value
**Group III**						
Before treatment	1.22 ± 0.250	0.0457	0.380	0.307, 0.453	10.69	0.000
After treatment	845 ± 0.189	0.0345	(<.001)			

CI: Confidence interval

The mean value of CRP levels in group II subjects with preterm delivery was 1.30 ± 0.201 (mean ± SD) and normal delivery was 1.037 ± 0.238 (mean ± SD). A significant difference was found in mean values of CRP levels (*P* =. 003) in preterm and term delivery in group II subjects. The mean values of CRP levels before treatment in group III subjects with preterm delivery was 1.25 ± 0.248 (mean ± SD) and normal delivery was 1.21 ± 0.255 (mean ± SD). There was no significant difference in mean values of CRP levels (*P* = 0.653) in preterm and term delivery subjects before treatment. The mean values of CRP levels after treatment in group III subjects with preterm delivery was 0.99 ± 0.167 (mean ± SD) and term delivery was 0.78 ± 0.162 (mean ± SD). A significant difference was found in mean values of CRP levels (*P* = .003) in preterm and term delivery subjects after treatment [Table 3]. The incidence of preterm and term delivery in group I was 8.3% and 41.7%, respectively (OR = 0.30, 95% CI = 0.134-0.674); and in group II was 31.7% and 18.3%, respectively (OR = 2.5,95% CI = 1.52-4.41). A significant difference was found in incidence of preterm and term delivery in group I and group II (*P* = 0.001) [Table 4]. The incidence of preterm and term delivery in group II was 31.7% and 18.3%, respectively (OR = 1.97, 95% CI = 1.14-3.39) and group III was 15.0% and 35.0%, respectively (OR = 0.49, 95% CI = 0.27-0.88). A significant difference was found in incidence preterm and term delivery in group II and group III (*P* = 0.010) [Table 5].

**Table 3:** CRP levels in group I, group II, and group III subjects (before and after periodontal therapy) with normal (term) and preterm delivery (*t*-test)

CRP	N	Mean ± SD	Std.error mean	Mean difference	95% CI of the difference	*t*	*P* value
Group I							
Term	25	0.674 ± 0.113	0.023				0.000
Preterm	5	0.912 ± 0.697	0.031	−0.238	−0.346, −0.129	−4.49	(<.001)
Group II							
Term	11	1.037 ± 0.238	0.071	−0.263			
Preterm	19	1.300 ± 0.201	0.046		−0.430, −0.096	−3.22	0.003
Group III							
Term	21	1.212 ± 0.255	0.055				
Preterm (Before treatment)	9	1.25 ± 0.248	0.082	−0.045	−0.252, 0.161	−454	0.65
Group IV							
Term	21	0.781 ± 0.162	0.035				
Preterm (After treatment)	9	0.995 ± 0.167	0.055	−0.214	−0.348, −0.080	−3.29	0.003

CI: Confidence interval

**Table 4:** Incidence of preterm and term delivery in group I and group II

Incidence	Preterm		Term		Total		Odds ratio (preterm/term)	95% CI
					
	Count	%	Count	%	Count	%		
Group								
I	5	8.3	25	41.7	24	40	300	0.134, 0.674
II	19	31.7	11	18.3	36	60	2.59	1.520, 4.417
Total	24	40.0	36	60	60	100	0.116	0.034, 0.0390

χ^2^ = 13.611; *P* = 0.001 sig

**Table 5:** Incidence of preterm and term delivery in group II and group III

Incidence	Preterm		Term		Total		Odds ratio (preterm/term)	95% CI
					
	Count	%	Count	%	Count	%		
Group								
II	19	31.7	9	15.0	28	46.7	1.974	1.148, 3.395
III	11	18.3	21	35.0	32	53.3	0.490	0.270, 0.887
Total	30	50.0	36	650	60	100	4.030	1.372, 11.83

χ^2^ = 6.696; *P* = 0.010 sig

## DISCUSSION

The results of this cohort study demonstrated the elevated CRP levels in pregnant women with periodontal disease compared to healthy controls and preterm delivery rate was higher in women with periodontal disease compared to control group. Significantly elevated CRP levels were found in subjects with periodontal disease. The results of the study are consistent with outcomes of recent investigations which reported an elevation of CRP levels in periodontitis patients.[9–12] Several underlying pathogenic mechanism for observation are possible. Periodontal pathogens do not induce only local inflammation and tissue destruction; they are involved in systemic increase in inflammatory and immune response.[13] Low levels of bacteremia, LPS, and other bacterial components may provide a stimulus for systemic inflammatory responses such as increased production of CRP due to activation of the cascade of inflammatory cytokines production by monocytes and other cells in periodontal tissues.[14]

There are very few studies to evaluate the association between periodontal disease and CRP levels in pregnant women. It has been shown that high CRP levels at the beginning of the pregnancy is associated with nearly two-fold increased risk if preterm delivery.[15] Moreover, CRP levels exceeding the threshold were associated with increased risk of preterm delivery.[2] In the present study, the mean value of reported CRP levels correlated with high incidence of preterm delivery. Subjects with preterm delivery have high mean CRP values compared to subjects with normal delivery. Periodontal treatment before 28 weeks of gestation resulted in significant decrease in mean CRP levels. Randomized trials of periodontal treatment have indicated that treatment of periodontal infections can significantly lower serum levels of CRP.[16–19] This study is in support to the reports of previous trails.[16–19] The data from this study showed that the incidence of preterm delivery was greater in subjects with periodontal disease compared to control group. This association was initially suggested by Offenbacher *et al*., 1996 and was further confirmed in various other studies.[20] Incidence of preterm delivery in treatment group was 19.0% compared to 31.7% in the study group. There was a significant reduction in incidence after treatment which showed that the treatment resulted in decrease in incidence of preterm delivery. Studies have indicated that periodontal treatment improves periodontal health and is safe,[21–23] and periodontal treatment reduces the incidence of PT/LBW rate.[24,17] Results obtained in the present study are consistent with previous studies.[17,24,25] In this study, the association observed between CRP and periodontal disease in pregnancy may or may not be causal. Periodontitis may increase CRP levels in pregnancy. CRP could amplify the inflammatory response through complement activation, tissue damage, and induction of inflammatory cytokines in monocytes,[26] and therefore, may mediate the association between periodontitis and adverse pregnancy outcomes. Periodontal treatment resulted in decrease in CRP levels and incidence of preterm delivery. This raises the possibility that CRP may mediate association between periodontal disease and preterm delivery. Further study on the maternal and fetal immune response to chronic oral infection and on placental pathology in women with periodontal disease has to be done to determine whether the relationship between periodontal disease and preterm is causal or simply associative.

However, since there has been no previous report on plasma CRP levels in pregnant women with and without periodontal disease and related incidence of preterm delivery in Indian population, the present study provides ground work data regarding the above correlation and promotes further studies in this direction.
